# Pituitary Mimic: Sellar Meningioma in a Patient Undergoing Fertility Therapy

**DOI:** 10.7759/cureus.85675

**Published:** 2025-06-10

**Authors:** Zeinab Alnahas, Anas Kartoumah, Talal Alomar, Kareem Masalkhi, Mohamad Horani

**Affiliations:** 1 Internal Medicine, Cairo University, Cairo, EGY; 2 Biomedical Sciences, University of South Florida, Tampa, USA; 3 Internal Medicine, Creighton University School of Medicine, Phoenix, USA; 4 Biology, Arizona State University, Phoenix, USA; 5 Internal Medicine, Chandler Regional Medical Center, Chandler, USA

**Keywords:** fertility treatment, gonadotropin-releasing hormone, meningioma, secondary adrenal insufficiency, sellar mass

## Abstract

Sex hormones play a crucial role in the development and growth of meningioma. It was observed that meningiomas increase in size and become more symptomatic during pregnancy. Similarly, the use of hormonal therapy, including gonadotropin-releasing hormone (GnRH) agonist, can be associated with an increased risk for meningioma. We reported a 45-year-old woman with a past medical history of hypothyroidism and mild hyperprolactinemia who received hormonal therapy for infertility, including GnRH. She has experienced a persistent headache and left retro-orbital pain with eye movement. Her MRI brain scan revealed a soft tissue mass involving the sellar and left cavernous sinus with a slight impingement of the optic chiasm and left optic nerve, concerning for meningioma or pituitary adenoma. Further laboratory workup showed secondary adrenal insufficiency, which was treated with replacement glucocorticoid therapy. Following tumor resection and histological examination, the diagnosis of sellar meningioma was confirmed. This case report shows a rare association of fertility hormonal treatment with sellar meningioma and how it is challenging to distinguish meningioma from other sellar masses, such as pituitary adenoma, based on clinical presentation and imaging studies.

## Introduction

Meningioma is the most common primary brain tumor arising from arachnoidal cells of the arachnoidal leptomeninges. According to the World Health Organization (WHO) grading system, meningiomas are classified into three grades based on their histopathological characteristics, with grade I defined as benign and representing about 80-90% of tumors, grade II defined as intermediate, and, less commonly, grade III defined as malignant [1]. Meningiomas usually develop along the dural lining of the venous sinuses in the cerebral convexity, parasagittal region, or sphenoid wing regions. Suprasellar or sellar meningiomas that arise near the sella occur in about 5-10% of all meningiomas [2]. Women have twice the increased risk of meningioma than men, which could be explained by the influence of sex hormones on the development and growth of meningioma. It was observed that meningiomas increase in size and become more symptomatic in the luteal phase of the menstrual cycle and during pregnancy [3]. Similarly, the use of hormonal therapy, including gonadotropin-releasing hormone (GnRH) agonist, can be associated with an increased risk for meningioma. This case report shows a rare association of fertility hormonal treatment with a sellar mass and possible differential diagnoses. This case is notable for its diagnostic complexity, specifically the overlap in imaging and clinical presentation between pituitary adenoma and sellar meningioma in a patient undergoing fertility therapy. The co-occurrence of hyperprolactinemia and secondary adrenal insufficiency, alongside imaging limitations due to potential pregnancy, highlights a rare and diagnostically challenging clinical scenario.

This work was previously presented as a meeting abstract (PMON223) at the Endocrine Society’s ENDO 2022 Annual Meeting on June 13, 2022.

## Case presentation

A 45-year-old obese woman with a past medical history of hypertension, prediabetes, hypothyroidism, and mild hyperprolactinemia presented to us with a two-week history of persistent and worsening periorbital headache associated with nausea, vomiting, and left retro-orbital pain with eye movement. She had undergone fertility treatment, intrauterine insemination, and hormonal therapy, including GnRH agonist, bromocriptine, and levothyroxine. At the time of presentation, she was taking levothyroxine (100 mcg daily) and bromocriptine (2.5 mg twice daily) as part of her ongoing endocrine management. She had a recent intrauterine insemination (IUI) one week before her presentation. On further questioning, she reported persistent fatigue and reduced appetite, which were nonspecific but possibly consistent with evolving adrenal insufficiency. She denied weakness, facial paresthesia, blurry/double vision, recent excessive weight gain/loss, or the use of any anticoagulation or antiplatelet medications. Additionally, she reported no increase in her ring or shoe size, menstrual disturbances, loss of peripheral vision, orthostatic dizziness, or skin pigmentation. Given her lab results, her presentation was consistent with central (secondary) adrenal insufficiency rather than peripheral (primary) adrenal disease.

Diagnostic assessment

**Figure 1:**
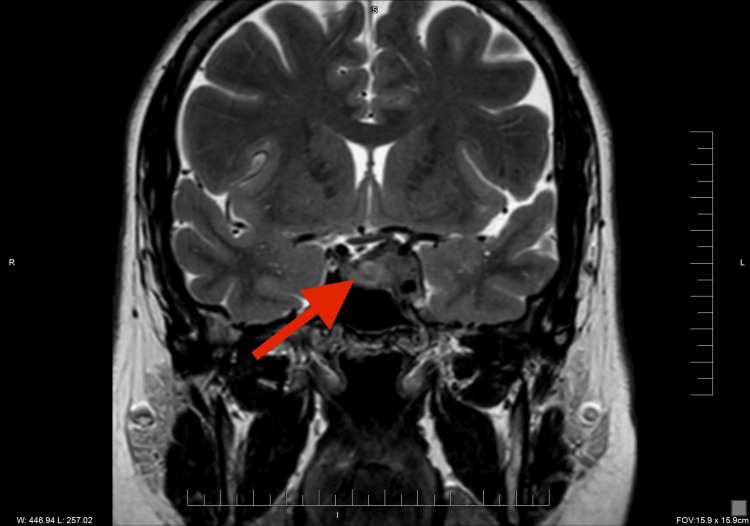
Coronal T2-weighted MRI Coronal view confirms asymmetric invasion of the left cavernous sinus (red arrow) and lateral displacement of the pituitary stalk; the optic chiasm is mildly compressed superiorly.

**Figure 2:**
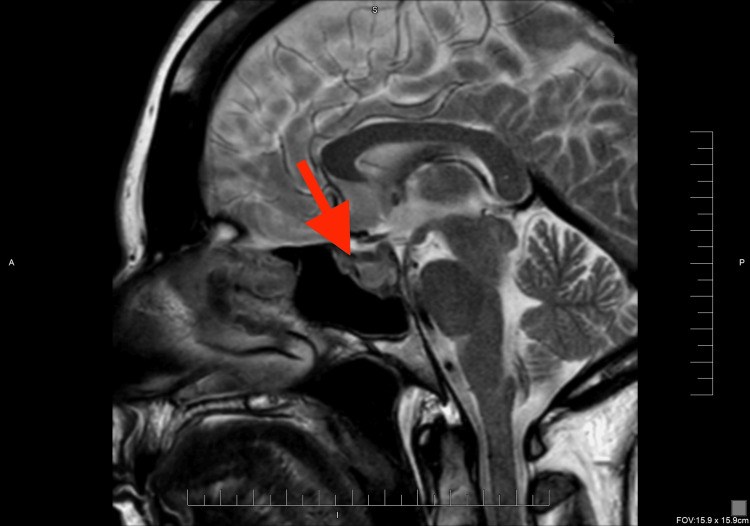
Sagittal T2-weighted MRI On T2 imaging, the same lesion appears heterogeneously hyperintense (red arrow), outlining its suprasellar extension and dural attachment—features compatible with meningioma rather than pituitary macroadenoma.

**Figure 3:**
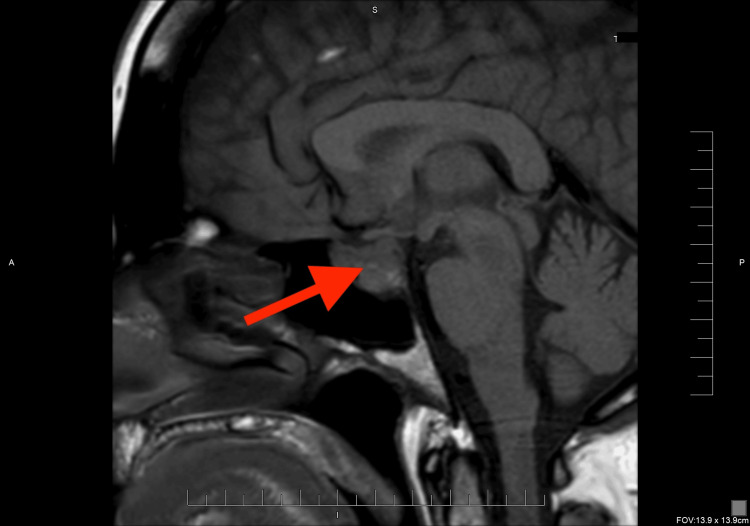
Sagittal non-contrast T1-weighted MRI Midline T1 sequence demonstrates an iso-to-hypointense 2.3 × 1.5 × 1.2 cm sellar mass (red arrow) that elevates the diaphragma sellae and indents the optic chiasm while extending into the left cavernous sinus.

An MRI brain scan without contrast was done due to potential pregnancy and revealed a 2.3 x 1.5 x 1.2 cm heterogeneous soft tissue mass involving the sellar and left cavernous sinus with a slight impingement of the optic chiasm and left optic nerve (Figures 1-3). These findings were consistent with meningioma or pituitary macroadenoma.

A comprehensive laboratory workup was conducted (Table 1), revealing elevated serum prolactin, low morning cortisol and adrenocorticotropic hormone (ACTH), and a subnormal response to the cosyntropin stimulation test, which confirmed the diagnosis of secondary adrenal insufficiency. Although TSH was within the normal range, this finding is of limited utility in evaluating for central hypothyroidism, as TSH may be inappropriately normal or even elevated in pituitary dysfunction. Free T4 was normal, effectively ruling out central hypothyroidism in this case. A cosyntropin stimulation test was performed to confirm adrenal insufficiency and to differentiate central (secondary) from primary adrenal causes, given the critically low baseline cortisol and low ACTH levels.

**Table 1:** Laboratory results

Test	Result	Reference Range	Interpretation
Serum prolactin	33.3 ng/mL	< 25 ng/mL	Elevated
TSH	2.11 mIU/L	0.5–5.0 mIU/L	Normal
Free T4	1.3 ng/dL	0.7–1.9 ng/dL	Normal
IGF-1	250 ng/mL	90–360 ng/mL (age 40–54)	Normal
Morning ACTH	6 pmol/L	10–60 pmol/L	Low
Morning cortisol	<0.2 mcg/dL	5–25 mcg/dL	Critically low
Cosyntropin response	Subnormal	Normal increase in cortisol expected	Confirmed secondary adrenal insufficiency

Treatment

The patient was treated with replacement therapy of short-acting glucocorticoid, hydrocortisone (20 mg PO, divided into two doses: 15/5 mg), and surgical resection of the sellar mass was scheduled. Six weeks later, the patient underwent an endonasal endoscopic transsphenoidal resection of the tumor. She received 100 mg IV of hydrocortisone before the operation, followed by 100 mg IV the night after. The glucocorticoid replacement dose was gradually tapered (150 mg IV on the first postoperative day, 100 mg IV on the second postoperative day) and then switched to her usual dose of 20 mg PO on the third postoperative day. The histopathological examination of the tumor tissue was composed of cells with meningothelial features embedded within fibrosis and sclerosis. Immunohistochemically, the tumor cells exhibited positive expression for antiepithelial membrane antigen (EMA) and somatostatin receptor 2A (SSTR2A). The MIB-1 labeling index for proliferative activity was <1%. These findings were consistent with meningioma, WHO grade 1.

Outcome and follow-up 

Following the complete surgical resection of the tumor, the patient was discharged on the fifth postoperative day without experiencing any complications. During the follow-up, there was no apparent evidence of tumor recurrence on her postoperative MRI imaging.

## Discussion

Sex hormone receptors, including estrogen and progesterone receptors, can be expressed in meningiomas and indicate these tumors' hormonal sensitivity. However, progesterone receptor expression is higher and can be found in 70-80% of meningiomas compared to the estrogen receptor expression, which can be lower than 10% [4]. The expression of progesterone receptors is correlated with the WHO grade, with a high rate of expression in benign WHO grade I tumors [5]. Activating these receptors by both endogenous and exogenous hormones, including hormonal therapy for fertility, can stimulate the growth of meningioma cells [6]. GnRH agonist is a widely used hormonal therapy for fertility among women with ovulation disorders that can stimulate ovulation, final follicular maturation, and oocyte release. It induces a mid-cycle LH surge, leading to progressively increased progesterone secretion. The use of GnRH agonist can be combined with intrauterine insemination to improve pregnancy outcomes [7]. Interestingly, this agonist can also stimulate pituitary GnRH receptors, leading to the proliferation of gonadotroph cells that may cause pituitary adenoma growth and apoplexy, especially gonadotrophinomas [8].

The location of meningiomas plays a crucial role in the clinical presentation, prognosis, and treatment options. Sellar meningiomas account for about 1% of sellar masses and usually present with visual disturbance, headache, and abnormalities of the pituitary hormones. However, it is difficult to clinically distinguish sellar meningioma from other sellar masses, including the more common pituitary adenoma that represents about 90% of all sellar masses [9]. An MRI scan with gadolinium is considered the standard diagnostic imaging for sellar masses due to its high spatial resolution and gadolinium enhancement. However, the use of gadolinium is contraindicated in pregnancy, severe renal impairment, and acute renal failure [10]. In our case report, the patient had an MRI scan without contrast due to potential pregnancy. It could not provide the exact origin and etiological diagnosis of the sellar mass, which was revealed after tumor resection and histological examination. The development of sellar mass during the use of GnRH agonist can be related to the growth of meningioma or pituitary adenoma, which can represent a diagnostic and therapeutic challenge.

Similarly, other rare hypothalamic lesions may present with overlapping endocrine symptoms and imaging features, complicating the differential diagnosis. For example, a recent case report described a tuber cinereum hamartoma causing secondary amenorrhea and hyperprolactinemia due to stalk compression, initially raising concern for pituitary pathology [11]. Although histologically distinct from meningiomas, such lesions highlight the importance of considering a broad differential diagnosis in hormonally active patients with sellar or suprasellar masses.

## Conclusions

This case highlights a rare but important association between fertility treatment using GnRH agonists and the development of sellar meningioma. The overlap in clinical and radiological features between meningiomas and pituitary adenomas can complicate diagnosis, particularly when MRI with contrast is contraindicated. Clinicians should maintain a high index of suspicion for intracranial tumors in patients presenting with persistent headaches and visual disturbances during or after hormonal therapy. Histopathological confirmation remains essential for definitive diagnosis and guiding appropriate management.

## References

[1] Louis DN, Perry A, Reifenberger G (2016). The 2016 World Health Organization Classification of Tumors of the Central Nervous System: a summary. Acta Neuropathol.

[2] Buetow MP, Buetow PC, Smirniotopoulos JG (1991). Typical, atypical, and misleading features in meningioma. Radiographics.

[3] Wiemels J, Wrensch M, Claus EB (2010). Epidemiology and etiology of meningioma. J Neurooncol.

[4] Commins DL, Atkinson RD, Burnett ME (2007). Review of meningioma histopathology. Neurosurg Focus.

[5] Maiuri F, Mariniello G, de Divitiis O (2021). Progesterone receptor expression in meningiomas: pathological and prognostic implications. Front Oncol.

[6] Hatiboglu MA, Cosar M, Iplikcioglu AC, Ozcan D (2008). Sex steroid and epidermal growth factor profile of giant meningiomas associated with pregnancy. Surg Neurol.

[7] Taheripanah R, Zamaniyan M, Moridi A, Taheripanah A, Malih N (2017). Comparing the effect of gonadotropin-releasing hormone agonist and human chorionic gonadotropin on final oocytes for ovulation triggering among infertile women undergoing intrauterine insemination: an RCT. Int J Reprod Biomed.

[8] Sasagawa Y, Tachibana O, Nakagawa A, Koya D, Iizuka H (2015). Pituitary apoplexy following gonadotropin-releasing hormone agonist administration with gonadotropin-secreting pituitary adenoma. J Clin Neurosci.

[9] Chaudhry SK, Raza R, Naveed MA, Rehman I (2021). Suprasellar meningiomas: an experience of four cases with brief review of literature. Cureus.

[10] Gatto F, Perez-Rivas LG, Olarescu NC (2020). Diagnosis and treatment of parasellar lesions. Neuroendocrinology.

[11] Ach T, Saafi W, Nouira S, Ben Abdelkrim A (2023). Secondary amenorrhea revealing a giant hamartoma of the tuber cinereum. Cureus.

